# Further support for the validity of the social appearance anxiety scale (SAAS) in a variety of German-speaking samples

**DOI:** 10.1007/s40519-021-01171-y

**Published:** 2021-06-03

**Authors:** Julia Reichenberger, Anne Kathrin Radix, Jens Blechert, Tanja Legenbauer

**Affiliations:** 1 Department of Psychology, Centre for Cognitive Neuroscience, Paris-Lodron University of Salzburg, Hellbrunnerstrasse 34, 5020 Salzburg, Austria; 2 LWL University Hospital Hamm for Child and Adolescent Psychiatry, Psychotherapy and Psychosomatic, Ruhr-University Bochum, Hamm, Germany

**Keywords:** Eating disorders, Social appearance anxiety, Questionnaire, Fear of evaluation

## Abstract

**Purpose:**

Eating disorders (ED) and social anxiety disorder are highly comorbid with potentially shared symptoms like social appearance anxiety (SAA) referring to a fear of being negatively evaluated by others’ because of overall appearance. SAA constitutes a risk factor for eating psychopathology and bridges between EDs and social anxiety disorder.

**Methods:**

The present studies examined internal consistency, factor structure, test–retest reliability, gender and age invariance, convergent validity and differences between individuals with and without an ED of a German version of the social appearance anxiety scale (SAAS) in four independent samples (*n*_1_* = *473; *n*_2_ = 712; *n*_3_ = 79; *n*_4_ = 33) including adolescents and patients with EDs.

**Results:**

Consistently, the SAAS showed excellent internal consistency (ωs ≥ 0.947) and a one-factorial structure. Convergent validity was shown via high correlations of the SAAS with social anxiety (e.g., social interaction anxiety *r* = 0.642; fear of negative evaluation *r*s ≥ 0.694), body image disturbance measures (e.g., shape concerns *r*s ≥ 0.654; weight concerns *r*s ≥ 0.607; body avoidance *r*s ≥ 0.612; body checking *r*s ≥ 0.651) and self-esteem (*r* = −0.557) as well as moderate correlations with general eating psychopathology (e.g., restrained *r*s ≥ 0.372; emotional *r* = 0.439; external eating *r* = 0.149). Additionally, the SAAS showed gender and age invariance and test–retest reliability after 4 weeks with *r* = 0.905 in Study 2 and was able to discriminate between individuals with and without an ED in Study 4.

**Conclusion:**

Hence, the German version of the SAAS can reliably and validly assess SAA in female and male adolescents or adults with or without an ED. Additionally, the SAAS might be used in a therapeutic context to especially target patient groups suffering from EDs with comorbid social anxiety.

**Level of evidence:**

Level III: Evidence obtained from cohort or case-control analytic studies.

## Introduction

Eating disorders (ED) such as anorexia nervosa, bulimia nervosa and binge eating disorder are highly prevalent in the general population [1] and are associated with physiological and psychological impairments in various domains [2]. Apart from that, EDs are also highly comorbid with social anxiety disorder (SAD), defined as a fear of social or performance situations in which a person is exposed to possible negative evaluation by others [DSM-5; 3]. To illustrate, Kaye et al. [4] reported that 20% of their ED patients were diagnosed with a comorbid lifetime SAD. Likewise, heightened levels of anxiety have been found to co-occur more frequently in families with EDs [5] and are hypothesized to impact the course of recovery [6, 7]. This suggests that common vulnerabilities and shared features might occur between both disorders. In a position paper, Pallister, Waller [8] even plead for a change in perspective. Based on eating-, weight- and shape-related cognitions, the authors interpret the psychopathology of EDs as safety behavior. Considering the great comorbidity with social anxiety [9], it is possible that constructs closely related to social evaluative anxieties trigger eating-related concerns, which in turn strengthen ED psychopathology. Hence, constructs such as fear of negative evaluation (FNE) or social appearance anxiety (SAA) may be particularly relevant when trying to understand this overlap.

To date, FNE has frequently been investigated in EDs: FNE scores are not only higher in individuals with an ED compared to healthy controls [10], but are also associated with ED severity, particularly on dimensions such as drive for thinness, bulimic attitudes, and body dissatisfaction [11]. In a longitudinal study DeBoer et al. [12] showed that the individual degree of FNE predicted the experienced level of future body dissatisfaction. This suggests that the extent of experienced FNE impacts one component of body image disturbance, which forms one of the core symptoms in EDs. The link between anxiety and body image disturbance has been strengthened by the demonstration that body size overestimations no longer significantly discriminated between patients with anorexia nervosa compared to controls after controlling for experienced anxiety during the experiment [13]. Hence, anxiety during assessment affected body image perception. Nevertheless, it remains unclear whether anxiety in general or more specific types of anxiety plays a key role in EDs.

Recent research has aimed at disentangling FNE from a subcomponent tapping into appearance-related FNE: SAA is defined as anxiety about being negatively evaluated by others because of one’s overall appearance, including, but not limited to, body shape. Indeed, introducing this separate concept provided more clarity on the shared mechanism between SAD and ED: Levinson et al. [14] demonstrated that SAA constitutes a shared risk factor between social anxiety and ED symptoms, whereas FNE was a risk factor for social anxiety symptoms only. Similarly, some SAA symptoms bridged between SAD and ED in a network analysis [15]. Manipulation of FNE and SAA in an experimental study with undergraduate women lends support to the notion that both anxiety factors produce differing effects: manipulations of FNE increased food intake, whereas manipulations of SAA increased body dissatisfaction [16]. Moreover, in a longitudinal study with undergraduate women, SAA prospectively predicted binge eating [17]. This contrasts with findings indicating that both anxiety dimensions are vulnerability factors for both social anxiety and ED symptoms: SAA predicted body dissatisfaction, bulimic symptoms, shape concern, and eating concern, whereas FNE selectively predicted drive for thinness and restraint in unselected individuals [18]. Nevertheless, separating general FNE from SAA might be important for studying shared and distinct effects of SAD and ED.

To assess SAA, Hart et al. [19] developed the social appearance anxiety scale (SAAS) as measure of “fear of situations in which one’s overall appearance may be evaluated”. The SAAS was created from measures of body image dissatisfaction, body dysmorphic disorder, and social anxiety. From the originally designed 17 items, 16 items showed sufficient psychometric properties and resulted in the final version of the SAAS. The SAAS demonstrated a one-dimensional factor structure using exploratory as well as confirmatory factor analysis with high internal consistency, convergent validity with measures of social anxiety and body image disturbances, divergent validity (e.g., by non-significant relationships with openness, agreeableness, conscientiousness, sympathy or ethnic identity) and test–retest reliability after 4 weeks in undergraduate samples [19, 20]. Moreover, the authors showed that the SAAS partially explains the link between social anxiety and negative body image in their structural equation model [19]. Even in patients with EDs, the SAAS showed good psychometric validity and significant relationships with body mass index (BMI), drive for thinness, body dissatisfaction, emotional problems (e.g., depression, anxiety) and interpersonal problems (e.g., suspiciousness, submissiveness) [21]. Patients with bulimia nervosa showed significantly higher SAAS scores compared to healthy controls [22] and SAA even seems to be capable among other factors to index the severity of bulimia nervosa [23]. Additionally, patients with bulimia nervosa and healthy controls differed with regard to their relationships between SAAS scores and eating pathology. In patients with bulimia nervosa, SAAS scores were related to higher global eating pathology and its subscale dietary restraint. In contrast, in healthy controls, SAAS scores were associated with higher global eating pathology and its subscales weight, shape, and eating concerns as well as higher BMI, and thus, tentatively more cognitive concerns instead of erratic eating behaviors [22].

Thus far, the SAAS has been translated and validated in Italian [24] and Turkish [25]. Again, the Italian version of the SAAS showed a one-factor structure using confirmatory factor analysis, high internal consistency, test–retest reliability after three weeks, convergent validity, as well as the ability to discriminate between adolescents with ED with or without SAD [24]. Moreover, the SAAS has been validated in under-examined populations like gay and bisexual men of color [26], or patients with systemic sclerosis [27]. However, up to now, neither the SAAS nor an alternative questionnaire for SAA (e.g., the fear of negative appearance evaluation scale which has not been used/studied frequently, see [28], or the social physique anxiety scale which is restricted to one’s physique like height, weight and muscle tone, see [29]) has been translated and validated in German.

Furthermore, apart from few studies [e.g., 24, 25] little is known about the psychometric properties of the SAAS in adolescents, although previous research showed that body dissatisfaction and appearance-related anxiety significantly increases during late childhood and youth [e.g., 30, 31]. More specifically, it has previously been shown that SAAS scores are higher in older compared to younger adolescents [24] but whether this trend continues into adulthood has not been tested yet. Similarly, little is known about the psychometric properties of the SAAS in female compared to male individuals. Previous research showed significant differences between both genders in that females exhibited higher scores than males [e.g., 24, 32]. As a result, the current study aimed at investigating the psychometric properties of a German translation of the SAAS, and to do so in two independent samples that together broaden the sample characteristics with regard to age and gender. Further, due to its importance for the clinical utility of the German version, we explored the validity of the scale in two additional samples of adolescents and adults suffering from diagnosed ED, as especially anorexia but also bulimia nervosa seem to have higher incidence rates in adolescence and young adulthood [1]. Similar to the original validation study [19], we predicted that the SAAS would constitute a one-factorial and internally consistent measure, with test–retest reliability. Moreover, we hypothesized the SAAS to be positively associated with measures of social anxiety (e.g., social interaction anxiety, FNE), body image disturbances (e.g., concerns about eating, shape and weight, as well as body checking and avoidance) and BMI, as well as negatively associated with global self-esteem. In contrast, we predicted that the SAAS would be associated with general measures of ED psychopathology (e.g., restrained eating, emotional and external eating) to a lesser extent. Additionally, as gender and age differences on the SAAS have previously been reported, we investigated the gender and age invariance of the scale and predicted higher scores on the SAAS for female compared to male participants. Using a four-study design with independent samples of healthy individuals and individuals with diagnosed EDs, we aimed to broaden sample characteristics to potentially increase the generalizability of the findings. Study 1 examined psychometric properties in female adolescents and young adults, whereas Study 2 broadened the sample characteristics with regard to age and gender, Study 3 and Study 4 examined adolescents and adults with a diagnosed ED, respectively.

## Methods

### Social appearance anxiety scale (SAAS): translation procedure

The social appearance anxiety scale [SAAS; 19] is a 16-item measure that assesses the anxiety of being negatively evaluated by others because of one’s overall appearance. Similar to the English version of the SAAS [19], items can be rated on a 5-point Likert-scale ranging from 1 (not at all) to 5 (extremely). The questionnaire encompasses items such as “I am concerned people would not like me because of the way I look.” or “I worry that my appearance will make life more difficult for me.” (see also Table 1). After re-coding of reversed item 1, a total sum score is calculated with higher scores indicating higher SAA.

**Table 1:** Items, factor loadings, and item statistics of the Social Appearance Anxiety Scale in Study 1

Item	CFA factor loadings subsample 2	EFA factor loadings subsample 1	*M*	SD	*r*_itc_	∝ if item deleted
1. I feel comfortable with the way I appear to others^a^ [Ich fühle mich wohl mit der Art, wie ich auf andere wirke.]^a^	0.671	0.596	2.90	1.04	0.609	0.971
2. I feel nervous when having my picture taken. [Ich fühle mich nervös, wenn ich mich fotografieren lasse.]	0.655	0.607	3.37	1.29	0.613	0.972
3. I get tense when it is obvious people are looking at me. [Ich werde angespannt, wenn deutlich ist, dass andere mich anschauen.]	0.709	0.752	3.33	1.25	0.717	0.970
4. I am concerned people would not like me because of the way I look [Ich bin besorgt, dass Menschen mich aufgrund meines Aussehens nicht mögen könnten.]	0.890	0.908	2.71	1.48	0.887	0.967
5. I worry that others talk about flaws in my appearance when I am not around [Ich sorge mich, dass andere in meiner Abwesenheit über Makel meines Äußeren reden.]	0.854	0.877	2.71	1.50	0.850	0.968
6. I am concerned people will find me unappealing because of my appearance [Ich bin besorgt, dass Menschen mich aufgrund meines Aussehens nicht ansehnlich finden.]	0.878	0.920	2.74	1.47	0.888	0.967
7. I am afraid that people will find me unattractive [Ich habe Angst, dass Menschen mich unattraktiv finden.]	0.851	0.882	2.79	1.44	0.849	0.968
8. I worry that my appearance will make life more difficult for me [Ich sorge mich, dass mir mein äußeres Erscheinungsbild das Leben erschweren wird.]	0.839	0.852	2.39	1.46	0.836	0.968
9. I am concerned that I have missed out on opportunities because of my appearance [Ich bin besorgt, dass mir aufgrund meines Aussehens Chancen entgangen sind.]	0.766	0.809	2.19	1.40	0.777	0.969
10. I get nervous when talking to people because of the way I look [Aufgrund meines Aussehens werde ich nervös, wenn ich mit Menschen spreche.]	0.828	0.843	2.11	1.32	0.819	0.969
11. I feel anxious when other people say something about my appearance [Ich fühle mich ängstlich, wenn andere Menschen etwas über mein Äußeres sagen.]	0.879	0.897	2.60	1.44	0.872	0.968
12. I am frequently afraid I would not meet others’ standards of how I should look [Ich habe häufig Angst, dass ich die Standards von anderen, wie ich auszusehen habe, nicht erfüllen könnte.]	0.856	0.898	2.56	1.48	0.857	0.968
13. I worry people will judge the way I look negatively [Ich sorge mich, dass Menschen mein Äußeres negativ beurteilen werden.]	0.937	0.923	2.66	1.45	0.911	0.967
14. I am uncomfortable when I think others are noticing flaws in my appearance [Ich fühle mich unwohl, wenn ich denke, dass andere Makel an meinem Äußeren bemerken.]	0.859	0.861	2.98	1.46	0.836	0.968
15. I worry that a romantic partner will/would leave me because of my appearance [Ich sorge mich, dass mich mein Partner aufgrund meines Aussehens verlassen wird/würde.]	0.697	0.741	2.27	1.47	0.696	0.971
16. I am concerned that people think I am not good looking [Ich bin besorgt, dass Menschen denken, dass ich nicht gut aussehe.]	0.906	0.906	2.59	1.40	0.885	0.968

German items are displayed in brackets. Response categories are (German translation in brackets)

*1 *not at all (trifft überhaupt nicht zu), *2* (trifft wenig zu), *3* (trifft etwas zu), *4* (trifft ziemlich zu), *5 *extremely (trifft völlig zu)

^a^Reverse-coded

Translation of the SAAS from English to German was conducted using the back-translation procedure: The SAAS was translated into German by the authors and then back translated into English by an English native speaker. To improve the quality of the German version, any differences between the original version and the re-translation were discussed by a committee consisting of the second and last author, alongside the native speaker who translated the scale. Because of reviewers’ comments pointing us to guidelines for questionnaire translation which recommend a combination of several methods [e.g., 33], we additionally tested the comprehension of the scale after initial data collection. To do so, 20 German-speaking females (age: *M* = 26.3, SD = 3.19 years old), who approximated the target population, rated each item on a 5-point Likert-type scale from 1 (= *do not understand at all*) to 5 (= *understand completely*). The overall understanding of the scale was very good with item comprehension of *M* = 4.58, SD = 0.69, ranging from 4.25 to 4.90. Thus, no revisions were made to the originally translated items and earlier collected results are reported.

### Measures for convergent validity

#### Brief fear of negative evaluation-revised (BFNE-R): Study 1,2,3

The BFNE-R [34, German version: 35] assesses fear and distress related to negative evaluation from others. Its 12 items are rated on a 5-point Likert-scale from 1 (not at all characteristic of me) to 5 (extremely characteristic of me). The sum score ranges from 12 to 60 with higher scores indicating greater FNE.

#### Social interaction anxiety scale (SIAS): Study 2

The SIAS assesses symptom severity for social anxiety disorder [German version: 36, 37]. This instrument comprises 20 items, rated on a 5-point Likert-scale from 0 (not at all) to 4 (very much). Higher sum scores indicate higher social interaction anxiety with a cutoff of 34 for sensitivity.

#### Eating disorder examination-questionnaire (EDE-Q): Study 1, 2, 3

The EDE-Q [38, German version: 39] was used to measure ED psychopathology in the past 28 days. The scale consists of 22 items coded from 0 (no days/not at all) to 6 (every day/markedly) that can be classified into four subscales.^1^

#### Body checking and avoidance questionnaire (BCAQ): Study 1, 3

The BCAQ [41] is a 27-item instrument and assesses body related checking and avoidance, as well as reassurance seeking on three subscales. Items can be answered on a 4-point Likert scale ranging from 1 (not at all true) to 4 (very true). An example item encompasses “I wear clothes that cover my whole body, even in the summer”. The BCAQ offers good convergent and divergent validity.

#### Multidimensional body-self relations questionnaire (MBSRQ): Study 2

The MBSRQ [German version: 42] assesses body image attitudes. Its 34 items are rated on a 5-point Likert-scale ranging from 1 (definitely disagree/never) to 5 (definitely agree/very often). The current study used three subscales of the MBSRQ that are associated with overall appearance. The Appearance Evaluation subscale measures feelings of physical attractiveness with seven items. Higher means indicate more positive feelings and satisfaction with appearance. The Appearance Orientation subscale measures the extent of investment in one’s appearance with 12 items. higher means indicate higher emphasis on appearance and engagement in extensive grooming behaviors. The Overweight Preoccupation subscale measures anxiety about one’s weight with four items. Higher means indicate higher anxiety.

#### Self-esteem: Study 2

Self-esteem was assessed with the single item ‘I have high self-esteem’ scored from 1 (= not at all true of me) to 7 (= true of me) with higher scores indicating higher global self-esteem [similar to 43].

#### Dutch eating behavior questionnaire (DEBQ): Study 2

The DEBQ [44, German version: 45] measures the extent of the three eating styles restrained, external and emotional eating (10 items each). Its items are rated from 1 (never) to 5 (very often) and higher mean scores indicate higher extent of the respective eating style.

### Data analysis

The current studies aimed at testing the following psychometric measures: (i) factorial structure, (ii) internal consistency, (iii) convergent validity, (iv) test–retest reliability, (v) measurement invariance with regard to gender and age, and (vi) differences between individuals with and without an ED.

To evaluate the factorial structure, the total sample of Study 1 (*N* = 473) was randomly divided into two subsamples (subsample 1: *n* = 237; subsample 2: *n* = 236). Based on post-hoc power considerations, the whole sample was large enough to be split, to conduct an exploratory factor analysis in subsample 1 (1:10 item-participant ratio yields a sample size requirement of 160 participants) and a confirmatory factor analysis in subsample 2 (sample size requirements based on [46] suggesting fair fit or close fit with regard to root mean square error of approximation (RMSEA) would yield 128 or 160 participants, respectively). Subsamples did not differ with regard to age (*t*_(471)_ = 0.265, *p* = 0.791), or BMI (*t*_(409.9)_ = 1.16, *p* = 0.248). In subsample 1, an exploratory factor analysis was applied and factor structure was tested with a principal component analysis and the number of components was determined by a parallel analysis and Velicer’s revised minimum average partial (MAP) test using the SPSS syntax by [47]. All SAAS items were below critical limits for skew <|2.0| and kurtosis <|7.0|. In addition, factor loadings and item total correlation (r_it_) scores were calculated for item selection. In subsample 2, a confirmatory factor analysis was computed with Amos 27 [48] to test the one-factor structure of the SAAS. Maximum likelihood estimation was used, fixing the factor loading of item 1 to 1. According to the recommendations of [49], model fit was evaluated with three fit indices: the comparative fit index (CFI), with 0.90 ≤ CFI < 0.95 indicating acceptable fit and CFI ≥ 0.95 indicating good fit, the root mean square error of approximation (RMSEA), with 0.05 < RMSEA ≤ 0.08 indicating acceptable fit and RMSEA ≤ 0.05 indicating good fit, and the standardized root mean square residual (SRMR), with 0.08 < SRMR ≤ 0.10 indicating acceptable fit and SRMR ≤ 0.08 indicating good fit.

Internal consistency was determined with McDonald’s ω because of the Likert-type scale using the OMEGA macro for SPSS [50].

To assess convergent validity, SAAS scores were correlated with measures of social anxiety (e.g., BFNE-R, SIAS) and body image disturbance (EDE-Q subscales, BCAC, MBSRQ) as well as general eating psychopathology (restraint EDE-Q subscale, DEBQ), self-esteem and BMI.

Test–retest reliability was assessed by Pearson’s correlation and intraclass correlation coefficient (ICC) with an absolute agreement, two-way mixed-effects model.

In Study 2, we evaluated whether the SAAS varies between female (*n* = 599) and male (*n* = 113) participants as well as different age groups. Two different age groups were built with late adolescence (age range in the current sample 16–24 years, *n* = 378) and young/middle adulthood (age range 25–50, *n* = 289). Measurement invariance was tested at the three levels configural, metric and scalar invariance and model fit changes were examined using recommendations by [51, 52]: A ΔCFI of less than 0.01 would be indicative of metric invariance. Scalar invariance would be obtained when ΔCFI < 0.01 in addition to a ΔRMSEA < 0.015 or ΔSRMR < 0.030. In case of gender and age invariance, differences between these groups were reported in Study 2. In addition, differences between age groups of ED patients were analyzed by comparing the young adolescent sample of Study 3 to the late adolescent/young adult sample of Study 4.

Additionally, an independent samples *t* test was calculated to compare an adult sample with and without EDs in their extent of SAA.

### Study 1: validation of the SAAS in youth and early adulthood

#### Participants and procedure

Participants^2^ were recruited via advertising posts on Facebook, blogs and online threads and completed the questionnaires online via the online platform EvaSys. Questionnaire completion took approximately 30 min, participation was anonymized, and all questions were mandatory in order to continue. However, participants could withdraw from participation at any time. Data were collected from April to October 2016. A total of 477 female participants provided complete data.^3^ Among those, four participants had to be excluded because of invalid sociodemographic data on height. The resulting 473 participants had a mean age of *M* = 21.6 years (*SD* = 2.71; 14–25) and a mean BMI of *M* = 21.9 kg/m^2^ (*SD* = 4.86; 13.9–59.8 kg/m^2^). All participants had female gender, most of them were German (93.4%) and 6.6% were classified as other nationalities. Participants could win one out of three 20 € Amazon vouchers. This study was carried out in accordance with the recommendations of and approved by the ethics committee of the medical faculty of the Ruhr-University Bochum (15-5500-BR). All subjects gave written informed consent in accordance with the Declaration of Helsinki. Parents or legal guardians were not obliged to provide written informed consent for the non-adult participants aged 14 years or older.

Initial checks for data quality revealed that all responses were in the range of plausible scores. To provide robustness, additional analyses were calculated excluding nine participants who did not show any variability in their item responses.

## Results

### Exploratory factor analysis: Subsample 1

**Fig. 1:**
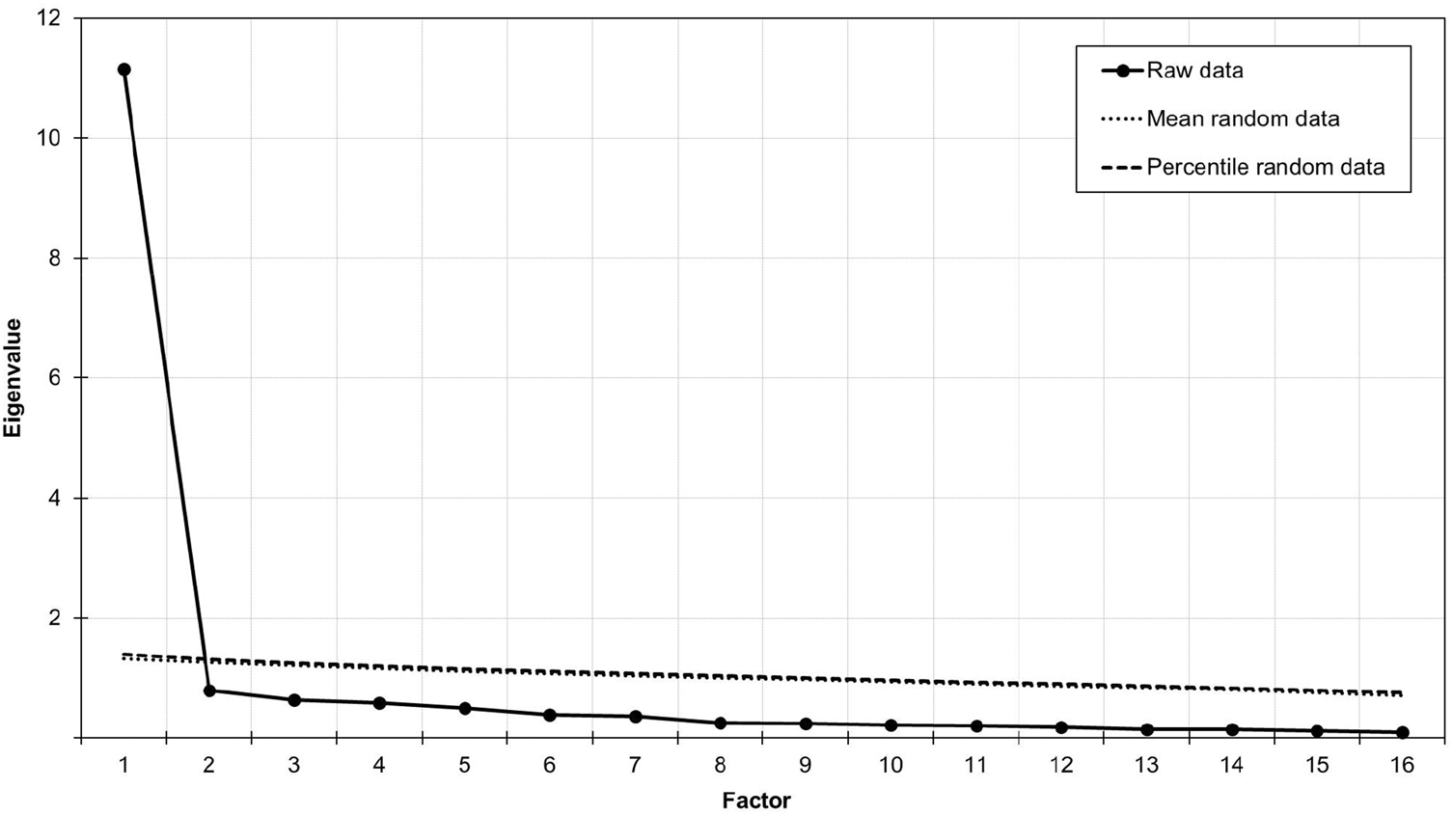
Scree plot and eigenvalues of the parallel analysis in Study 1

The Kaiser–Meyer–Olkin measure of sampling adequacy (KMO = 0.962) and Bartlett’s test of sphericity (X^2^_(120)_ = 4261, *p* < 0.001) indicated that the data was appropriate for exploratory factor analysis. Both the MAP test (smallest average 4th power partial correlation: 0.0024) and parallel analysis (Fig. 1) suggested a one factor structure, explaining 69.8% of variance. All factor loadings were higher than 0.596 (Table 1).

### Confirmatory factor analysis: Subsample 2

The initial model revealed mixed results with a poor fit according to RMSEA = 0.111, acceptable fit to CFI = 0.924 and good fit to SRMR = 0.035. However, modification indices showed high covariances between error terms. Indeed, items showed partial overlap due to similar wordings in the German translation (e.g., item 2, 3: “Ich fühle mich nervös…” and “Ich werde angespannt…”, referring to feeling nervous and tense; item 5, 6, 7: “Ich sorge mich, dass…” and “Ich bin besorgt, dass…”, referring to being worried and being concerned; and both items 8, 9: “…dass Menschen mich… finden”). Thus, we allowed correlations between four error couples (2 & 3; 5 & 6; 6 & 7; 8 & 9) which resulted in the final model with better fit: Although RMSEA (0.073) indicated acceptable fit, SRMR (0.027) and CFI (0.969) indicated good fit. All factor loadings were greater than 0.655 (all *p*s < 0.001) (Table 1).

### Internal consistency

Internal consistency in the whole sample was excellent with McDonalds’ ω = 0.973.

### Convergent validity

Descriptives of the questionnaires and Pearson correlations of the whole sample are reported in Table 2. As expected, the SAAS was positively correlated with measures of social anxiety, i.e. higher FNE, and body image disturbances, i.e., higher eating, weight and shape concern, body checking and body avoidance. In addition, higher SAAS was moderately to highly correlated with higher restraint, however, to a smaller extent than to body image disturbances, and correlation with BMI was positive, but low.^4^

**Table 2:** Descriptive statistics of continuous study variables and correlations with SAAS scores in Study 1

Variable	*M*	SD	Range	α	*r*	*p*
Social appearance anxiety scale	42.9	18.7	16–80	–	–	–
Brief fear of negative evaluation scale-revised	39.2	13.0	12–60	0.964	0.746	< 0.001
Body checking and avoidance questionnaire-avoidance	1.84	0.705	1.00–4.00	0.895	0.735	< 0.001
Body checking and avoidance questionnaire-checking behavior	2.00	0.670	1.00–3.75	0.861	0.708	< 0.001
Eating disorder examination questionnaire						
Eating concern	1.50	1.61	0–6	0.881	0.700	< 0.001
Shape concern	2.86	1.83	0–6	0.935	0.755	< 0.001
Weight concern	2.46	1.83	0–6	0.879	0.727	< 0.001
Restraint	2.19	1.83	0–6	0.884	0.550	< 0.001
Body mass index (BMI) (kg/m^2^)	21.9	4.86	13.9–59.8	–	0.101	0.029

## Discussion

Results of Study 1 showed a one-factor structure, an excellent internal consistency and convergent validity of the German version of the SAAS in younger participants. In line with previous research, the SAAS showed stronger correlations with social anxiety and body image disturbance, but weaker relationships with restraint eating and BMI making the application of complementary measures (e.g., other questionnaires for social anxiety with a different focus; or other cognitively influenced eating styles like external eating) worthwhile. As Study 1 comprised only female participants and adolescents as well as young adults, Study 2 aimed at replicating current results and extending them by the following aspects: First, sample characteristics were broadened by including male participants to assess measurement invariance for gender and examine the previously reported gender differences on the SAAS [e.g., 24, 32]. Second, we included a broader age range to assess measurement invariance for age because of previous findings of age differences on the SAAS [24] and potentially important developmental trajectories of SAA and body dissatisfaction [30, 31]. Third, we aimed at assessing test–retest reliability after 4 weeks. Fourth, we applied additional measures for convergent validity potentially broadening the importance of the SAAS to aspects of general self-esteem or more general eating psychopathology.

### Study 2: validation of the SAAS in a gender and age extended sample

#### Participants

Participants were recruited via student mailing lists at universities in Germany and Austria, flyers as well as word of mouth and partook in the study ‘self-compassion, body image and eating behavior’ from March to June 2019. The study used an unselected sample without specific inclusion or exclusion criteria. To measure test–retest reliability, two administrations were conducted with survey 1 including the SAAS and measures of validity (among other questionnaires not of relevance for the current study) and survey 2 (4 weeks later) with the SAAS and additional measures of validity. A total of 714 participants from survey 1 provided complete data on sociodemographic details and the SAAS; however, two had to be excluded because of invalid body height entries. The resulting 712 participants (84.1% female) had a mean age of *M* = 27.9 years (*SD* = 10.4; 16–88) and mean BMI of *M* = 22.8 kg/m^2^ (*SD* = 5.61; 14.2–65.4 kg/m^2^). Participants had *M* = 16.4 (*SD* = 3.29) years of education and were mainly German (65.9%), Austrian (27.4%), Swiss (0.7%) and Others (6.0%). Among those, a subsample of 327 participants (89.3% female) completed survey 2, with a mean age of *M* = 27.2 years (*SD* = 9.70; 16–88) and mean BMI of *M* = 22.6 kg/m^2^ (*SD* = 6.10; 14.9–61.0 kg/m^2^).

#### Procedure

After recruitment, participants completed the first online survey via Limesurvey with a fixed order of questionnaires and a completion time of 21 min on average. Participants were informed that they give their consent for study participation by clicking ‘next’ online. Every question required a response in order to continue, however, participants had the option to withdraw from study participation any time and without any disadvantages. Participants were asked to provide their email address and were instructed about a follow-up survey. Four weeks later participants were contacted and asked about participation in the second online survey with an average completion time of 45 min. Both the first and the second survey included the SAAS to assess test–retest reliability, apart from that, convergent validity measures were spread across both surveys (survey 1: DEBQ, self-esteem, BMI; survey 2: BFNE-R, SIAS, MBSRQ). On average, mean time between administration of both surveys was *M* = 29.9 days (*SD* = 5.27 days). Data were anonymized and combined between both assessments by a self-created code of the participants and participants were informed about this procedure. Three 50 € prizes were raffled among participants who completed the first survey, and two additional 50 € prizes among participants who completed the second survey. Additionally, participants had the option to receive information about the study purpose and results via email. Procedures were approved by the local ethics committee of the University of Salzburg.

Data quality was assessed as follows: although we refrained from tracking IP addresses and setting cookies, multiple participation was avoided by participants having to indicate a self-created code and no duplicate codes were determined. All answers of the SAAS items were in the plausible range of the response format. Although completion time for the SAAS differed between participants, there were no outliers, defined as z-scores >|3|, on the lower end of the completion time. Nevertheless, we additionally calculated the analyses excluding five participants without timing data and 12 participants who needed less than 30 s to complete the SAAS (control analysis 1). Additionally, we re-analyzed the data excluding 15 participants who showed no variance in the responses of the SAAS items (control analysis 2).

## Results

### Confirmatory factor analysis, gender, and age invariance

Applying the model obtained in Study 1 revealed similar results with an acceptable fit according to RMSEA = 0.077, and good fit to CFI = 0.961 and SRMR = 0.030. All factor loadings were greater than 0.611 (all *p*s < 0.001).

To examine measurement invariance across female and male participants, we used multi-group confirmatory factor analysis. Model fit changes between the configural invariance model (CFI = 0.958, RMSEA = 0.056, SRMR = 0.046) and the metric invariance model (CFI = 0.957, RMSEA = 0.054, SRMR = 0.059) indicated gender invariance (ΔCFI = 0.001, ΔRMSEA = 0.002, ΔSRMR = 0.013). In addition, model fit changes between the scalar model (CFI = 0.952, RMSEA = 0.056, SRMR = 0.068) and the metric model indicated gender invariance (ΔCFI = 0.005, ΔRMSEA = 0.002, ΔSRMR < 0.001). Women reported higher scores in the SAAS (*M* = 39.4, *SD* = 16.0) compared to men (*M* = 32.7, *SD* = 12.4; *t*_(189.9)_ = 5.03, *p* < 0.001).

Similarly, to examine measurement invariance across late adolescence and young/middle adulthood, we used multi-group confirmatory factor analysis. Model fit changes between the configural invariance model (CFI = 0.956, RMSEA = 0.057, SRMR = 0.030) and the metric invariance model (CFI = 0.956, RMSEA = 0.055, SRMR = 0.031) indicated age invariance (ΔCFI < 0.001, ΔRMSEA = 0.002, ΔSRMR = 0.001). In addition, model fit changes between the scalar model (CFI = 0.952, RMSEA = 0.056, SRMR = 0.032) and the metric model indicated age invariance (ΔCFI = 0.004, ΔRMSEA = 0.001, ΔSRMR = 0.001). Late adolescents reported higher scores in the SAAS (*M* = 40.6, *SD* = 15.7) compared to young/middle aged adults (*M* = 36.7, *SD* = 15.4; *t*_(665)_ = 3.15, *p* = 0.002).

### Internal consistency and test–retest reliability

Internal consistency was excellent with McDonalds’ ω = 0.966 in survey 1 and McDonald’s ω = 0.961 in survey 2. Four weeks test–retest reliability was excellent with *r*_(327)_ = 0.905 and an ICC = 0.901 [95% CI 0.879; 0.920].

### Convergent validity

Descriptives and correlations of the assessed questionnaires can be seen in Table 3. The SAAS was again highly associated with measures of social anxiety like FNE as well as social interaction anxiety. Additionally, the SAAS was highly correlated with measures of body image disturbance like concerns about eating, weight and shape as well as negative body image attitudes. In addition, the SAAS negatively correlated with higher self-esteem. In contrast, correlations with measures such as external, emotional or restrained eating and BMI were rather of low to medium size, although significant.^5^

**Table 3:** Descriptive statistics of continuous study variables and correlations with SAAS scores in Study 2 in survey 1 and 2

Variable	*M*	SD	Range	α	*r*	*p*
Social appearance anxiety scale in survey 1	38.3	15.7	16–80	–	–	–
Survey 2 associations						
Brief fear of negative evaluation scale-revised	37.3	12.0	13–60	0.949	0.694	< 0.001
Social interaction anxiety scale	27.3	14.8	1–71	0.921	0.642	< 0.001
Eating disorder examination questionnaire						
Eating concern	0.80	1.10	0–4.8	0.839	0.608	< 0.001
Shape concern	1.91	1.57	0–6	0.920	0.682	< 0.001
Weight concern	1.54	1.54	0–6	0.848	0.607	< 0.001
Restraint	1.22	1.43	0–6	0.852	0.409	< 0.001
Multidimensional body-self relations questionnaire						
Appearance evaluation	3.37	0.95	1–5	0.927	−0.726	< 0.001
Appearance orientation	3.10	0.71	1.3–4.9	0.874	0.415	< 0.001
Overweight preoccupation	2.20	1.03	1–5	0.777	0.517	< 0.001
Survey 1 associations						
Dutch eating behavior questionnaire						
External eating	3.18	0.69	1–5	0.861	0.149	< 0.001
Emotional eating	2.56	0.94	1–5	0.932	0.439	< 0.001
Restrained eating	2.58	0.91	1–5	0.925	0.372	< 0.001
Self-esteem	4.50	1.52	1–7	–	–0.557	< 0.001
Body mass index (BMI) (kg/m^2^)	22.8	5.61	14.2–65.4	–	0.089	0.018

## Discussion

Similar to Study 1, results of Study 2 provided support for the one-factor structure in a confirmatory factor analysis. In addition, the scale exhibited internal consistency, convergent validity with measures of social anxiety, body image disturbance and negative body image attitudes, as well as general disordered eating behavior. Furthermore, results showed test–retest reliability of the SAAS after 4 weeks and the scale exhibited invariance across gender and age groups. Study 3 aimed at extending the validation efforts in examining internal consistency and convergent validity in an adolescent sample with much higher psychopathology (inpatient sample), particularly in the ED domain. This was done as previous research has shown differential relationships between SAAS scores and erratic eating behaviors versus more cognitive concerns in individuals with EDs compared to healthy controls [e.g., 22] and because of few studies investigating the psychometric properties of the SAAS in an adolescent sample with EDs.

### Study 3: validation in an adolescent sample with ED

#### Participants and procedure

A total of 108 participants were screened during inpatient treatment in the LWL university hospital for child and adolescent psychiatry in Hamm. Data were collected between November 2015 and November 2020. Because of missing ED diagnosis or diagnosis information, 21 patients were excluded, and additional 8 patients because of completely missing SAAS scores. Missing data on single SAAS items were replaced by the mean. The resulting 79 patients (95% female) had a mean age *M* = 15.0 years (*SD* = 1.34) and a mean BMI of *M* = 17.4 kg/m^2^ (*SD* = 5.82; 12.7–55.6 kg/m^2^). All participants received written and oral information about the study and provided written informed consent (if under the age of 18, also from their parents) prior to participation. After agreement, participants received the mentioned set of paper–pencil questionnaires they filled out in their rooms. Participants did not receive any financial compensation for their participation.

## Results

### Internal consistency

Internal consistency was excellent in the present study with McDonald’s ω = 0.957.

### Convergent validity

As expected and similar to the other studies, the SAAS was positively correlated with measures of social anxiety, i.e., higher FNE and body image disturbances, i.e., higher concerns about eating, shape and weight (see Table 4). In addition, the SAAS was positively associated with higher body checking and avoidance behavior. In contrast, the SAAS exhibited a moderate to high positive correlation with restrained eating and a small to moderate positive correlation with BMI.

**Table 4:** Descriptive statistics of continuous study variables and correlations with SAAS scores in Study 3

Variable	*M*	SD	Range	*r*	*p*
Social appearance anxiety scale	51.9	17.3	17–80	–	–
Brief fear of negative evaluation scale-revised	45.0	13.4	12–60	0.768	< 0.001
Body checking and avoidance questionnaire-avoidance	2.15	0.721	1–3.83	0.694	< 0.001
Body checking and avoidance questionnaire-checking behavior	2.23	0.658	1.08–3.83	0.733	< 0.001
Eating disorder examination questionnaire					
Eating concern	2.81	1.59	0–5.8	0.683	< 0.001
Shape concern	3.85	1.79	0–6	0.754	< 0.001
Weight concern	3.38	1.80	0–6	0.711	< 0.001
Restraint	3.06	2.01	0–6	0.534	< 0.001
Body mass index (BMI) (kg/m^2^)	17.4	5.82	12.7–55.6	0.272	0.016

## Discussion

Even within this sample of adolescents diagnosed with an ED in inpatient treatment, the SAAS exhibited good psychometric properties: Similar to Study 1 and 2, the results of Study 3 provide support for the internal consistency as well as convergent validity of the SAAS. To provide evidence for the clinical utility of the SAAS, a comparison between patients with EDs and healthy controls seems necessary. Thus, in Study 4, we aimed to a) provide support for the ability of the SAAS to differentiate between matched individuals with and without an ED and b) replicate the internal consistency in a sample with adults diagnosed with an ED. Moreover, based on the findings of Study 2, we aimed at examining differences in age groups of young adolescent and late adolescent/young adult ED patients. As inpatient treatment might have an influence on ED psychopathology, Study 4 explicitly sampled patients with an ED before an inpatient treatment.

### Study 4: validation in an adult sample with ED

#### Participants

A total of 33 female patients with an ED were recruited before inpatient treatment at a large clinic in Southern Germany specialized in treating EDs. Patients were diagnosed with a structured clinical interview [54] and met the respective DSM-5 criteria with 26 (72.7%) patients fulfilling the criteria for anorexia nervosa, 8 (24.2%) for bulimia nervosa, and 1 (3.0%) for binge eating disorder. The patients had a mean age of *M* = 24.3 years (*SD* = 10.4) and a mean BMI of *M* = 18.8 kg/m^2^ (*SD* = 5.81; 12.2–44.8 kg/m^2^). To compare SAAS scores between ED patients and healthy controls, a matched subsample of Study 2 was drawn with the exclusion criteria of male gender and reporting of former psychological disorders. As a result, ED patients and healthy controls did neither differ in BMI (*t*_(32.1)_ = 0.005, *p* = 0.996), age (*t*_(41.3)_ = -0.734, *p* = 0.467), nor years of education (*t*_(64)_ = 0.922, *p* = 0.360).

### Procedure

Patients participated and were diagnosed in a former bigger project including naturalistic, experimental, and psychometric data (not of relevance for the current manuscript). Subsequent to this project, patients were contacted and asked to complete the SAAS. Written informed consent was obtained from all participants and also from their parents if under the age of 18. The SAAS questionnaire was completed online via Limesurvey and data were collected from April 2020 to July 2020 by the first author. To do so, each participant received a specific code and only the first author would have been able to de-anonymize the data. All items required a response to continue, but participants could withdraw from participation at any time. Participants were not compensated for the additional completion of the SAAS. The overall project was approved by the ethics committee of the University of Salzburg (13/2016) and the medical review board at the University of Munich (396-16).

## Results

### Internal consistency

Internal consistency was excellent in the ED group with McDonald’s ω = 0.947.

### Differences between ED patients and healthy controls

As expected, ED patients exhibited higher SAAS scores compared to healthy controls, *t*_(64)_ = −6.41, *p* < 0.001, Cohen’s *d* = 1.58), with a *M* = 60.3 (*SD* = 12.9) in ED patients and *M* = 38.3 (*SD* = 14.9) in matched controls.

### Comparison of age groups

To check for differences between age groups, as obtained in Study 2, we compared young adolescent participants of Study 3 to late adolescent / young adult participants of Study 4. As expected, both ED groups significantly differed with regard to age *t*_(32.4)_ = −5.12, *p* < 0.001, Cohen’s *d* = 1.25), but did not significantly differ in BMI *t*_(109)_ = −1.19, *p* = 0.239). Early adolescents (*M* = 51.9, *SD* = 17.3) reported lower SAAS scores compared to individuals in late adolescence/early adulthood (*M* = 60.3, *SD* = 12.9), *t*_(79.5)_ = −2.81, *p* = 0.006, Cohen’s *d* = 0.547.

## Discussion

Again, the SAAS exhibited good psychometric properties with support for the internal consistency in an adult ED sample. Moreover, SAAS scores can differentiate between individuals with and without an ED; however, because of the small sample size of the study, results have to be taken as preliminary and should be replicated in a larger group of patients with an ED. Again, significant differences were found between age groups, further suggesting that late adolescence/early adulthood seems an especially vulnerable time period for SAA.

## Overall discussion

The present study examined the SAAS as one potential instrument to shed light on similarities and differences regarding the fear of being negatively evaluated between social anxiety and eating psychopathology. Therefore, we validated a German version of the SAAS, a measure for assessing anxiety about being negatively evaluated based on one’s appearance, in four independent samples. The German SAAS was demonstrated to be a one factorial, internally consistent measure in unselected individuals and individuals with a diagnosed ED. The scale exhibited convergent validity via high correlations with measures of social anxiety and body image disturbances, as well as small to moderate correlations with general eating psychopathology. In addition, the SAAS showed gender and age invariance and test—retest reliability after 4 weeks, indicating that the SAAS assesses a stable construct across time, gender and age. Importantly, the SAAS seems able to discriminate between individuals with and without an ED and, therefore, shows clinical utility.

In line with previous studies [e.g., 19, 24], the SAAS showed excellent internal consistency in all four samples (McDonald’s ω ranging between .947 and .973). Thus, the results provide support for the 16-item, German version of the SAAS. Moreover, the obtained high test–retest reliability after 4 weeks seems in accordance with previous studies [19, 24] and demonstrates the stability of SAA over this time period.

Convergent validity of the German SAAS was obtained in all three studies. The SAAS showed high relationships with social anxiety measures like FNE and social interaction anxiety. In addition, the SAAS related to body image disturbances in that higher SAAS scores correlated with greater body checking and avoidance behavior and body image attitudes like less feelings of physical attractiveness, higher investment in one’s appearance and greater anxiety about one’s weight. Moreover, higher SAAS was related to less global self-esteem and greater general eating psychopathology like concerns about eating, weight and shape, as well as restrained, emotional, and external eating. Hence, results are in line with previous studies examining the convergent validity of the SAAS [18–20, 24] and extend them by testing additional measures for general eating psychopathology. Therefore, the SAAS seems especially capable of examining the overlap between social anxiety and EDs. Previous studies already found support for SAA as a link between social anxiety and negative body image [14, 18, 19]. Although support for divergent validity has been found in a recent study [20], future studies might profit from focusing on measures assessing divergent validity to provide more conceptual clarity of the SAAS. Findings with regard to BMI differed according to the sample studied: Whereas in unselected individuals (Study 1 and 2), there was a low, positive correlation between BMI and SAAS scores, the correlation was moderate and positive in ED patients (Study 3). Although results of Study 3 are comparable to an earlier study examining a mixed ED sample [21], they are also in contrast to a study in patients with bulimia nervosa suggesting no significant relationship between BMI and SAAS scores [22]. In contrast, [22] also found moderate correlations in healthy controls, whereas our correlations were of a smaller magnitude, potentially due to the younger age of our participants.

Importantly, the present studies examined a variety of different samples, ranging from adolescents to young adults and from healthy participants to individuals with EDs. Similar to a previous study [24], the SAAS showed gender invariance, in that the one-factor structure upholds in female and male participants, making the scale similarly applicable to both genders. In line with previous studies [24, 32], we showed that females scored higher on the SAAS compared to men. Similarly, the SAAS showed age invariance within the two age groups of late adolescence and young/middle adulthood, making the scale similarly applicable to these groups. Additionally, we demonstrated that unselected individuals in their late adolescence exhibited higher SAAS scores compared to young/middle aged adults. Simultaneously, in our ED patients, individuals in their late adolescence/young adulthood exhibited higher SAAS scores compared to early adolescents. These results are in line with the finding that older compared to younger adolescents exhibited higher SAAS scores [24] and suggest that there may be a critical period for SAA and related phenomena like body image disturbances during late adolescence/early adulthood. However, future studies need to sample higher rates of adolescents with an equally distributed age range to examine measurement invariance across these groups and conduct longitudinal examinations to depict trajectories of SAA. In sum, the SAAS seems reliable and valid for individuals varying in age, gender, and eating psychopathology. Moreover, the SAAS seems to discriminate between individuals with and without an ED. Apart from patients diagnosed with an ED, future studies might profit from examining a clinical sample with SAD as well as a sample with comorbid social anxiety and ED [e.g., 24] to further examine the role of SAA for the etiology and maintenance of these disorders.

### Limitations and outlook

Although the present studies involved samples with diverse characteristics, future studies might profit from a greater sample of individuals with diagnosed EDs to replicate the psychometric properties and assess differences and similarities in samples with pure or comorbid disorders. In particular, the clinical sample of Study 3 consisted of an adolescent population with most patients being diagnosed for the first time. Hence, it remains uncertain whether SAA may also change over the course and history of an ED such as in chronic patient groups. Similarly, it remains for future research to assess the predictive power of current SAA for future ED symptoms in a longitudinal study. In addition, across all four groups our participants represented a rather young population from adolescence to young adulthood. It is possible that SAA may also be subject of change across the lifespan depending on developmental tasks. Additionally, the SAAS was initially translated using a method of back-translation only, although later testing of the comprehension of the scale also revealed good results. However, since the start of data collection best-practice guidelines for translation procedures of body image questionnaires suggest the application of a 5-stage procedure including also committee discussions [55].

In the future, the SAAS may be used as a promising instrument to investigate not only the overlap between social anxieties and eating psychopathology, but rather the relationship or potential impact of the fear of being negatively evaluated on treatment outcome in patients with EDs. As already documented in the literature, trait anxiety has a negative impact on treatment outcome [6, 7]. In addition to findings that show SAA correlates with different dimensions of eating psychopathology, the SAAS may be a potential instrument to investigate its role in treatment outcome. Similarly, knowledge of SAA may be used in the therapeutic context. First, intervening in SAA might be worthwhile, especially in individuals with comorbidity of social anxiety and ED. To illustrate, a previous study showed that repetitive thinking may increase SAA and thus constitute a maintenance mechanism for symptoms [56]. However, it remains for future research to explore the capability of body image interventions (e.g., exposure) to also reduce SAA. Second, not considering SAA in gold standard interventions like video feedback might hinder the effect of the treatment [e.g., as has been shown for physical appearance anxiety; 57].

### Conclusions

To conclude, the current studies demonstrated that the SAAS is an internally consistent, one-factorial measure that is associated with related concepts of social anxiety and body image disturbance as well as to a lesser extent general eating psychopathology. The SAAS seems to be an easily applicable measure in a variety of sample: in younger and older age, in healthy individuals but also patients with EDs, and similarly in females and males. Thus, we conclude that the SAAS is a useful and psychometrically sound tool for the investigation of SAA.

## What is already known on this subject?

Eating and social anxiety disorders exhibit high comorbidities, potentially due to fear of negative evaluation. More specifically, fear of negative evaluation because of one’s overall appearance—social appearance anxiety (SAA)—has been shown to relate to eating disorder psychopathology like binge eating and body image disturbances. Hence, SAA seems a promising candidate to contribute to the high comorbidity between both disorders. However, up to now, no valid and reliable questionnaire exists to assess SAA in German-speaking populations and there is still little research on the construct of SAA.

## What this study adds?

The present studies validate a German version of a questionnaire assessing SAA, to provide the first valid and reliable measure for German-speaking populations. Moreover, the present studies further support the function of SAA as a bridge between social anxiety and eating disorder psychopathology and demonstrate good psychometric properties in especially vulnerable populations like adolescents with or without eating disorders. Results suggest that measuring SAA in patients with social anxiety or eating disorder might provide important knowledge for therapeutic interventions.

## Data Availability

Not applicable.

## Footnotes

1 However, the four-factor structure has partially been reproduced for the German version of the EDE-Q only, with research supporting a three-factor structure with weight and shape concern combined into one factor [e.g., 40].

2 Part of the sample has previously been used in a different publication by Radix et al. [53].

3 Only participants who responded to each item of the SAAS were used, as either each item was completed or missing. Hence, data could not have been imputed.

4 Exclusion of the participants who did show variance in their responses to SAAS items did not change any of the results significantly.

5 Again, all results remained similar when excluding either participants with no variability in their responses to SAAS items (control analysis 1) or participants who needed less than 30 s to complete the SAAS (control analysis 2).

## Declarations

### Availability of data and material

Data will be made available on request.
